# High-concentration (8%) capsaicin patch for chronic postoperative neuropathic pain: A systematic review of randomised controlled trials

**DOI:** 10.1177/20494637251396094

**Published:** 2025-11-19

**Authors:** Megan Niven, Morgan Inwood, Patrice Forget

**Affiliations:** 1 School of Medicine, Medical Sciences and Nutrition, University of Aberdeen, Aberdeen, UK; 2 School of Medicine, Medical Sciences and Nutrition, Institute of Applied Health Sciences, Epidemiology Group, University of Aberdeen, Aberdeen, UK; 3 Department of Anaesthesia, NHS Grampian, Aberdeen, UK; 4 Pain and Opioids After Surgery (PANDOS) Research Groups, European Society of Anaesthesiology and Intensive Care, Brussels, Belgium; 5 IMAGINE UR UM 103 Montpellier University Anesthesia Critical Care, Emergency and Pain Medicine Division, Nîmes University Hospital, Nîmes, France

**Keywords:** chronic postoperative neuropathic pain, high-concentration (8%) capsaicin patch, systematic review

## Abstract

**Background:**

Chronic postoperative neuropathic pain is a common and sometimes disabling problem. Mainstay pharmacological management involves gabapentinoids, tricyclic anti-depressants and serotonin and norepinephrine reuptake inhibitors. Past this, guidance is limited. There is good evidence for the use of high-concentration capsaicin patch in non-operative causes of neuropathic pain. This systematic review aimed to evaluate the evidence base for the high-concentration (8%) capsaicin patch for postoperative neuropathic pain.

**Methods:**

We carried out a systematic search of 4 databases (Ovid MEDLINE, Embase, Cochrane Library and https://ClinicalTrials.gov) from inception to 3rd July 2025 to identify randomised controlled trials investigating the effectiveness of high-concentration capsaicin patch for postoperative neuropathic pain. The primary outcome was pain improvement, with adverse events being the secondary outcome. Study selection was performed independently by two reviewers using the Rayyan platform.

**Results:**

487 studies were identified. After screening, only one randomised controlled trial on 46 participants met inclusion criteria. The high-concentration capsaicin patch did not significantly improve postoperative neuropathic pain compared to an inactive placebo patch. However, the certainty of evidence was graded as very low using the Grading of Recommendations Assessment, Development and Evaluation (GRADE) approach. We also found 2 ongoing trials without published results.

**Conclusion:**

This systematic review identified a clear gap in the literature regarding the use of high-concentration capsaicin patches for chronic postoperative neuropathic pain. High-quality studies are needed to expand the existing evidence base. Based on our findings, we propose several recommendations to guide future research in this area.

## Background

Pain is an unpleasant emotional and sensory experience generated by thalamocortical networks in response to ascending signals from nociceptors.^1^ The International Association for the Study of Pain (IASP) defines chronic neuropathic pain as chronic pain persisting ≥3 months and occurring because of damage to the somatosensory nervous system.^2–4^ Chronic postoperative neuropathic pain is a significant clinical problem affecting up to 54% of patients, often difficult to manage.^5–9^ Amitriptyline is often thought of as first line, but studies suggest it only provides minor pain relief.^10^ Duloxetine, gabapentin and pregabalin have a better evidence base but side effects, for example, dizziness, somnolence and oedema limit their use.^11–13^ Pregabalin and gabapentin also have significant addiction potential. Furthermore, most evidence refers to painful diabetic neuropathy or postherpetic neuralgia, and not postoperative neuropathic pain. As a last resort, strong opioid medicines like morphine, oxycodone and tramadol can be prescribed with good efficacy^14^; however, side effects are common and can be significant, for example,, respiratory depression and constipation. Also, physical dependence can develop even with short-term use. Lastly, each of these medications also has drug interactions while topical capsaicin has no known drug interactions.^15^ Topical lidocaine may have a role but this is probably limited.^16,17^

There is moderate-certainty evidence that high-concentration capsaicin patches should be used for postherpetic neuralgia, HIV‐neuropathy and painful diabetic neuropathy.^18–21^ Low-concentration topical capsaicin has a very low-certainty evidence base.^22^ Given that high-concentration capsaicin is a TRPV1 agonist capable of inducing temporary neurolysis of nociceptor terminals by causing excessive calcium influx and subsequent calpain activation, it may be highly relevant in the management of postoperative neuropathic pain.^23,24^ This is the first PROSPERO-registered, PRISMA-reported, RCT-only synthesis, evaluating the evidence base for the use of high-concentration topical capsaicin in postoperative neuropathic pain, with the Revised Cochrane Risk of Bias Tool for Randomised Trials (RoB 2) and GRADE assessment.^25–27^ This gap analysis directly informs two ongoing Phase III trials enrolling over 800 patients.^28,29^

## Methods

### Registration

This systematic review has been prepared in line with the Preferred Reporting Items for Systematic Reviews and Meta-Analyses (PRISMA) statement.^30^ The protocol was published in the International Prospective Register of Systematic Reviews (PROSPERO CRD42023483171) during piloting of the study selection process (22/11/2023).

### Eligibility criteria

This systematic review aims to identify randomised controlled trials (RCTs) evaluating the effectiveness of high-concentration (8%) capsaicin for postoperative neuropathic pain. There were no post hoc changes to the a priori protocol (see Table 1). Both published and ongoing works were sought regardless of date of publication (or commencement of study) or language. Only full-text articles were included.

**Table 1.** Study eligibility criteria.

	Inclusion	Exclusion
Patient	Chronic postoperative neuropathic pain	Neuropathic pain not caused by surgery, for example, diabetes and postherpetic
Intervention	High-concentration (8%) capsaicin	Low-concentration capsaicin
Comparators	Placebo low-concentration capsaicin	
Outcome	Primary = change in pain score from baseline to follow-up; secondary = adverse events	

### Search strategy

**Figure 1.**
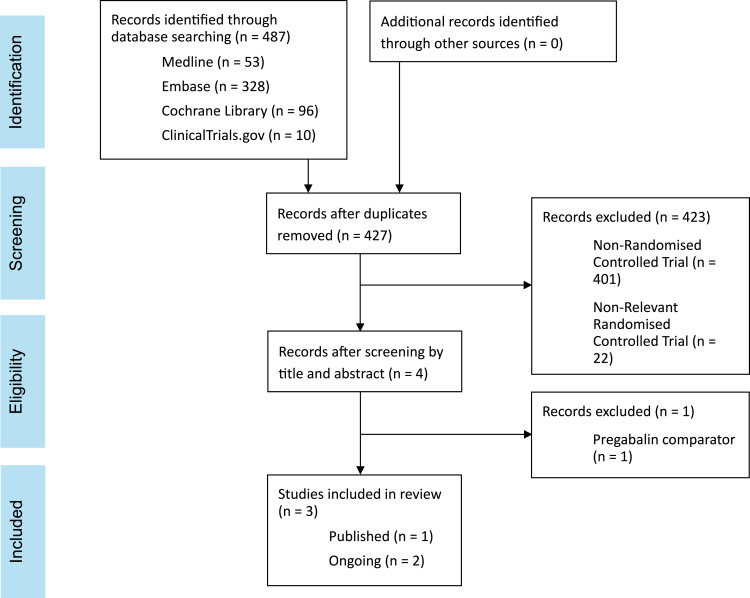
PRISMA flow chart showing the results of the systematic search of Ovid MEDLINE, Embase, Cochrane Library and https://ClinicalTrials.gov carried out from database inception to 3rd July 2025.

See Figure 1 and Appendix 1. Google Scholar was searched for grey literature to access any applied knowledge. Bibliographies were screened for additional publications.

### Analysis

Study selection, data extraction and bias assessment were performed independently by two reviewers (MN and MI). Disagreements were discussed between reviewers until a consensus was reached. Selection was facilitated by Rayyan, a web-based automated screening tool which stores references, presents the title and abstract of each study in a standard format, highlights potential duplicates and allows reason for exclusion labels to be added to studies.^31^ The following data items were extracted: author(s), year published, country of study, cohort size, gender, mean age, population, comparator, follow-up time, pain scoring and adverse events. The RoB 2 and GRADE approach were used to assess the evidence base.

### Data synthesis

Meta-analysis was not possible given only one study was identified. A narrative synthesis was done.

## Results

The search strategy identified 487 studies but only one was eligible for inclusion in this systematic review^32^ (see Figure 1).

### Primary outcome: Change in pain score between baseline and follow-up

Bischoff et al.^32^ found that there was no significant difference in summed pain intensity between high-concentration capsaicin and placebo patch treatments at 1, 2 and 3 months after patch application (mean numerical rating scale (NRS) difference [95% CI]: 5.0 [0.09 to 9.9], *p* = .046, −1.7 [−6.4 to 3.1], *p* = .48, 3.6 [−3.1 to 10.2], *p* = .29, respectively).

### Secondary outcome: Adverse events

Bischoff et al.^32^ found a significant difference in skin reactions between high-concentration capsaicin (74%) and placebo patch (30%) (p = .006). There was no significant difference in other secondary outcomes (see Appendix 3).

This study included 46 adult patients with severe (NRS ≥5) chronic neuropathic pain >6 months after inguinal herniorrhaphy, 42 (95%) of which were male. The mean age was 53 years. Pain was evaluated at baseline and at 1, 2 and 3 months after patch application using a summed pain intensity score comprising pain at rest, during movement and during palpation. A summary of the study characteristics is provided in Appendix 2.

Risk of bias was determined as high for this study, as shown in Table 2. The randomisation process was methodologically sound. Groups were computer-generated, concealed and potential confounding factors were evenly distributed as shown in the baseline characteristics table. However, issues were found in the four remaining domains of RoB 2.

**Table 2.** Revised Cochrane risk of bias tool for randomised trials.

	Risk of bias domains					
	D1	D2	D3	D4	D5	Overall
Bischoff et al. (2014)	Low risk	Some concerns	High risk	Some concerns	High risk	High risk

Domains:

D1: Bias arising from the randomisation process.

D2: Bias due to deviations from intended intervention.

D3: Bias due to missing outcome data.

D4: Bias in measurement of the outcome.

D5: Bias in selection of the reported result.

Even though the capsaicin patches and the inactive placebo patches were visually identical, 75% of patients were able to correctly report their allocation 1 month after patch application due to local side effects, despite having topical local anaesthetic cream (EMLA) applied 60 min prior to patch application by the research nurse. The physician administering the patches, although not further involved in the study, may also have been aware of allocation due to irritant substance transfer during patch handling. Four participants allocated were not analysed. One withdrew prior to treatment; one required early patch removal due to pain and two were lost to follow-up. Non-protocol interventions were balanced between groups. Other analgesia was only permitted if participants had been on a fixed dose for >4 weeks prior to study entry and for the duration of the study. Two patients began treatment with other analgesia during the study, so their data was only included prior to this. Adherence was not a consideration given only one application of the patch was required.

Selective data presentation was a significant issue. The primary outcome data are presented in a line graph, but mean absolute values are given only for baseline. There are also missing data for four of the seven secondary outcomes, namely, anxiety and depression, pain-related sleep disturbance, catastrophizing behaviour and neuropathic pain components.

Two eligible ongoing phase 3 trials, NCT05997979^28^ and NCT04967664,^29^ were identified but no results are currently available for either.

## Discussion

We have reviewed the literature on the use of high-concentration (8%) capsaicin patch for chronic postoperative neuropathic pain. This is the first PROSPERO-registered, PRISMA-reported, RCT-only synthesis with RoB 2 and GRADE assessment. The one trial identified showed the high-concentration capsaicin patch did not significantly improve postoperative neuropathic pain compared to an inactive placebo patch. However, there are issues with the study. It was under powered due to dropouts. A power calculation was carried out a priori which provided a recruitment target of 50 for a power of 0.9. Given there were 4 dropouts, only 46 participants were included in the study. The significance threshold was set at <0.01 to account for the use of primary and multiple secondary outcome measures assessed at several time points. There was ineffective blinding giving rise to placebo effects and self-reporting bias. There is also selective data presentation and missing data.

The recent Lancet review by Soliman et al.^21^ comparing various pharmacology and non-invasive neuromodulation agents for neuropathic pain recommended high-concentration capsaicin patches as second line, with low-dose capsaicin cream recommended if patches are not available. Seven studies, with a high risk of bias, were included for high-concentration capsaicin. The combined number needed to treat (NNT) was 13.2, while the number needed to harm (NNH) was 1129.3. Although the NNT was higher than other agents like tricyclic antidepressants (4.6) and serotonin and norepinephrine reuptake inhibitors (7.4), the NNH for these agents was greater, 17.1 and 13.9, respectively. Given their lower risk, high-concentration capsaicin patches may be a very useful treatment option for older adults with multiple comorbidities or polypharmacy, so long as the area of neuropathic pain has not broken, infected or inflamed skin and can be covered by the maximum of four patches allowed by the manufacturer. They should be used with caution in patients with uncontrolled hypertension as application may cause a transient increase in blood pressure due to the burning sensation.

### Recommendations for future research

The utilisation of an active placebo may help overcome ineffective blinding. This may be achieved by using low-concentration capsaicin or natural weak TRPV1 agonists such as piperine from black pepper or gingerol from ginger.^33,34^ Since capsaicin patch therapy is a painful treatment, researchers should expect dropouts. Bischoff et al.^32^ suggest that approximately 8% of participants may drop out, offering useful insights for determining a suitable study sample size. It has been shown that absence of allodynia predicts a good response to the capsaicin patch in patients with neuropathic pain from postherpetic neuralgia.^35^ Therefore, recording the presence or absence of associated features of neuropathic pain (e.g. altered sensation, burning, tingling and allodynia) at baseline would be useful to determine if this relationship also applies to postoperative neuropathic pain. Generalisability can be improved by enrolling participants who have undergone different surgical procedures and reporting comorbidities at baseline, which also allows for controlling potential confounders. Lastly, exploring secondary outcomes such as quality of life measures (e.g. SF-36^36^), patient willingness to repeat treatment, and willingness to reduce or stop other analgesics because of the capsaicin patch would aid understanding of effectiveness and tolerability.

## Conclusions

Although only one study has been identified, this paper stands as a methodologically rigorous review of the evidence base regarding high-concentration capsaicin patches for chronic postoperative neuropathic pain. Foremost, we have identified an important gap in the knowledge base. Currently, capsaicin patches in the UK are only approved for use in postherpetic neuralgia, precluding their use in people with chronic postoperative neuropathic pain. Our review demonstrates that this is an omission based on very little evidence, and a large number of patients may therefore be deprived of a potentially effective treatment for chronic neuropathic pain. High-quality studies are needed to expand the existing evidence base and draw robust conclusions about efficacy. Based on our findings, we propose several recommendations to guide future research on the use of capsaicin patches for chronic postoperative neuropathic pain.

## Supplemental Material


Supplemental material - High-concentration (8%) capsaicin patch for chronic postoperative neuropathic pain: A systematic review of randomised controlled trials
Supplemental material for High-concentration (8%) capsaicin patch for chronic postoperative neuropathic pain: A systematic review of randomised controlled trials by Megan Niven, Morgan Inwood and Patrice Forget in British Journal of Pain

## Data Availability

Data is provided within the manuscript or supplementary information files.*
