# Supplementary material for: High-concentration (8%) capsaicin patch for chronic postoperative neuropathic pain: A systematic review of randomised controlled trials
Source: Br J Pain. 2025 Nov 19:20494637251396094. Online ahead of print. doi: 10.1177/20494637251396094 (PMC12629964; doi:10.1177/20494637251396094)
Supplement: Supplemental material - High-concentration (8%) capsaicin patch for chronic postoperative neuropathic pain: A systematic review of randomised controlled trials [file sj-pdf-1-bjp-10.1177_20494637251396094.pdf]

Appendix

Appendix 1: Example of the search strategy

Appendix 2: Summary of the characteristics of the included studies

Appendix 3: Secondary outcome

Ovid MEDLINE(R) ALL <1946 to July 03, 2025>

1 Capsaicin/ 11669

2 Qutenza.mp. 47

3 Pain, Postoperative/ 53431

4 Postsurgical pain.mp. 1939

5 Postoperative neuropathic pain.mp. 72

6 Postsurgical neuropathic pain.mp. 42

71 or 2 11684
83 or 4 or 5 or 6 54206
97 and 8 53

Embase Classic+Embase <1947 to 2025 July 03>

1 capsaicin/ 25266

2 Qutenza.mp. 273

3 postoperative pain/ 113493

4 Postsurgical pain.mp. 2720

5 Postoperative neuropathic pain.mp. 131

6 Postsurgical neuropathic pain.mp. 69

71 or 2 25270

8 3 or 4 or 5 or 6 114206

97 and 8 328

Cochrane Library <1996 to 2025 July 03>

ID Search Hits

#1 capsaicin 1681

#2 qutenza 53

#3 postoperative pain 72785

#4 postsurgical pain 2788

#5 Postoperative neuropathic pain 886

#6 Postsurgical neuropathic pain 215

#7 {OR #1-#2} 1687

#8 {OR #3-#6} 73475

#9 {AND #7-#8} 96

Clinicaltrials.gov <2000 to 2025 July 03>

1 post surgical neuropathic pain or postoperative pain or postsurgical pain or neuropathic pain

2 capsaicin or qutenza

3 1 and 2 10

Appendix 2: Summary of the characteristics of the included studies

<!--Col Count:10-->Author	Country	Cohort size	Female/ male	Mean age	Population	Intervention	Comparator	Outcome(s)	Results
Bischoff et al. (2014)	Denmark	46	4/42	53	Patients ≥18 years with severe	High concentration (8%) capsaicin	Inactive placebo patch.	**Primary** Comparisons of summed pain	**Primary** The mean difference [95%

<!--Col Count:10-->Author	Country	Cohort size	Female/ male	Mean age	Population	Intervention	Comparator	Outcome(s)	Results
					(numerical rating scale [0-10] ≥5)<br>unilateral persistent inguinal post-herniorrhaphy pain lasting >6 months.	patch.		intensity (numerical rating scale) differences between capsaicin and placebo treatments at 1, 2 and 3 months after patch application	CI] in summed pain intensity between capsaicin and placebo patch treatments at 1 month after patch application was 5.0 [0.09 to 9.9], p=0.046.
								**Secondary**<br>• Intraepidermal nerve fibre density<br>• Quantitative sensory function<br>• Anxiety and depression (hospital anxiety and depression	The mean difference at 2 months after patch application was −1.7 [−6.4 to 3.1], p=0.48.

<!--Col Count:10-->Author	Country	Cohort size	Female/ male	Mean age	Population	Intervention	Comparator	Outcome(s)	Results
								scale)<br>• Pain-related sleep interference (daily sleep interference scale)<br>• Catastrophizing behaviour (pain catastrophizing scale)<br>• Neuropathic pain components (Leeds assessment of neuropathic symptoms and signs pain scale)<br>• Skin reactions	
									The mean difference at 3

<!--Col Count:10-->Author	Country	Cohort size	Female/ male	Mean age	Population	Intervention	Comparator	Outcome(s)	Results
									months after patch application was 3.6 [−3.1 to 10.2], p=0.29.
									<u>**Secondary**</u> **Intraepidermal nerve fibre density**
									The mean difference [95% CI] in intraepidermal nerve fibre density in the capsaicin group from baseline to 1 month after patch application was -1.9 (0.1 to -3.9), p=0.32.
									The mean

<!--Col Count:10-->Author	Country	Cohort size	Female/ male	Mean age	Population	Intervention	Comparator	Outcome(s)	Results
									difference [95% CI] in intraepidermal nerve fibre density in the placebo group from baseline to 1 month after patch application was -0.6 (1.2 to -2.5).
									**Quantitative sensory function**<br><br>The mean difference [95% CI] in warmth detection threshold between capsaicin and placebo patch treatments at 1

<!--Col Count:10-->Author	Country	Cohort size	Female/ male	Mean age	Population	Intervention	Comparator	Outcome(s)	Results
									month after patch application was 0.4, p=0.52.<br><br>The mean difference [95% CI] in cool detection threshold between capsaicin and placebo patch treatments at 1 month after patch application was -0.1, p=0.96.<br><br>The mean difference [95% CI] in heat pain threshold between capsaicin and

<!--Col Count:10-->Author	Country	Cohort size	Female/ male	Mean age	Population	Intervention	Comparator	Outcome(s)	Results
									placebo patch treatments at 1 month after patch application was 0.0, p=0.91.<br><br>The mean difference [95% CI] in pressure pain threshold between capsaicin and placebo patch treatments at 1 month after patch application was -1.9, p=0.91.<br><br>The mean difference [95% CI] in suprathreshold heat pain

<!--Col Count:10-->Author	Country	Cohort size	Female/ male	Mean age	Population	Intervention	Comparator	Outcome(s)	Results
									perception between capsaicin and placebo patch treatments at 1 month after patch application was 0.0, p=0.56.
									**Skin reactions**<br><br>74% of capsaicin treated patients and 30% of placebo treated patients reported ≥1 skin reactions at the patch application site, p=0.006.

Appendix 3: Secondary outcome

<!--Col	Adverse events

Count:7-->	Sensory function	Skin reactions	Anxiety and depression	Pain-related sleep interference	Catastrophizing behaviour	Neuropathic pain components
Bischoff et al. (2014)	No significant difference in thermal thresholds, suprathreshold heat pain perception, and pressure pain thresholds between high concentration capsaicin and placebo patch treatments at one, two or three months after patch application.	74% of participants treated with high concentration capsaicin patch had a skin reaction, compared to 30% of placebo treated participants (p=0.006). These were defined as erythema, pain or a burning sensation.	No significant difference in mental state using the hospital anxiety and depression scale between high concentration capsaicin and placebo patch treatments at one, two or three months after patch application.	No significant difference in sleep quality using the daily sleep interference scale between high concentration capsaicin and placebo patch treatments at one, two or three months after patch application.	No significant difference in catastrophisation using the pain catastrophizing scale between high concentration capsaicin and placebo patch treatments at one, two or three months after patch application.	No significant difference in neuropathic pain features using the Leeds assessment of neuropathic symptoms and signs pain scale between high concentration capsaicin and placebo patch treatments at one, two or three months after patch application.
